# Plasma Concentrations of Benzylpenicillin and Cloxacillin in Infective Endocarditis—With Special Reference to Delayed Hypersensitivity Reactions

**DOI:** 10.3390/antibiotics14010056

**Published:** 2025-01-09

**Authors:** Malin Hägglund, Ulrika Snygg-Martin, Lars Olaison, Michael Stofkoper, Bert Ove Larsson, Magnus Brink

**Affiliations:** 1 Department of Infectious Diseases, Sahlgrenska University Hospital, Region Västra Götaland, Diagnosvägen 21, SE-41650 Gothenburg, Sweden; ulrika.snygg-martin@infect.gu.se (U.S.-M.); lars.olaison@medfak.gu.se (L.O.); magnus.brink@vgregion.se (M.B.); 2 Department of Infectious Diseases, Institute of Biomedicine, Sahlgrenska Academy, University of Gothenburg, Guldhedsgatan 10a, SE-40234 Gothenburg, Sweden; 3 Department of Infectious Diseases, Södra Älvsborgs Sjukhus, Region Västra Götaland, Samaritvägen 1, SE-50740 Borås, Sweden; mstofkoper@hotmail.com; 4 Department of Infectious Diseases, Norra Älvsborgs Länssjukhus, Regions Västra Götaland, Lärketorpsvägen 20, SE-46173 Trollhättan, Sweden; bert-ove.larsson@vgregion.se

**Keywords:** therapeutic drug, monitoring, infective endocarditis, beta-lactams, delayed hypersensitivity reaction, beta-lactam fever

## Abstract

Background: Current antibiotic regimens for infective endocarditis (IE) are effective but pose a high risk of delayed hypersensitivity reactions (DHR). Dose adjustments guided by therapeutic drug monitoring (TDM) could mitigate these risks while maintaining treatment efficacy. This study aimed to investigate the plasma concentration of benzylpenicillin and cloxacillin in patients with IE and explore associations between antibiotic concentrations and DHR. Methods: Plasma concentrations of benzylpenicillin and cloxacillin were measured as centre (midpoint concentrations between consecutive doses) and trough values during the first and third weeks of treatment in patients with IE. Patient characteristics and outcomes, including DHR, were documented. Results: A total of 55 patients were included, with 37 patients (67%) receiving benzylpenicillin and 18 (33%) receiving cloxacillin. The 90-day mortality rate was 3%. Both centre and trough concentration exhibited substantial interpatient variation for the two antibiotics, while intra-patient variability between weeks 1 and 3 remained low for most patients. Kidney function could explain, at best, 54% of the variation, and a multiple regression model including kidney function, body mass index, age, and albumin explained up to 68% of the variation for benzylpenicillin. There was no relation between high plasma concentration and the prevalence of DHR; conversely, we observed a tendency of low plasma concentrations in these patients. Conclusions: This study revealed significant interindividual variation in plasma concentrations for both studied penicillins. TDM might be useful in situations where concentrations are hard to predict, such as severe obesity or kidney failure. Additionally, we found no indication that high plasma concentrations are related to the prevalence of DHR.

## 1. Introduction

Infective endocarditis (IE) is a severe infection of the heart’s inner lining and valves, with an incidence of 5–11 cases per 100,000 person-years [1,2]. Treatment duration is long, typically lasting 4–6 weeks, and involves administration of antibiotics at higher doses than those used for most other infections [3]. The epidemiological pattern of IE has undergone significant changes over the last few decades. While it was once primarily a disease affecting young and middle-aged adults with underlying rheumatic or congenital heart disease, it now predominantly affects individuals with risk factors such as degenerative heart valve disease, prosthetic heart valves, cardiac implantable electronic devices (CIEDs), and those undergoing hemodialysis. Staphylococci have become the most common causative agents, followed by viridans group streptococci [4,5]. In Sweden, the prevalence of methicillin-resistant Staphylococcus aureus (MRSA) is low, with over 99% of Staphylococcus aureus blood culture isolates being methicillin-sensitive. Streptococcus species also show high susceptibility to benzylpenicillin, resulting in a favorable resistance profile. Therefore, national guidelines recommend beta-lactam antibiotics, such as benzylpenicillin and cloxacillin, as first-line treatments for infective endocarditis [6,7].

It is well established that the efficacy of penicillins and other beta-lactam antibiotics closely correlates to the time when the free (unbound) concentration of the drug remains over the minimal inhibitory concentration (MIC) of the bacteria targeted. Free concentrations exceeding four times the MIC do not provide an additional bactericidal effect [8,9]. Treatment strategies for IE continue to rely heavily on clinical experience and older retrospective studies [10].

Current standardized treatment regimens with fixed high doses of beta-lactam antibiotics for most patients achieve high cure rates but are also associated with a relatively high incidence of delayed hypersensitivity reactions (DHR). Up to one third of patients experience DHR between treatment days 15 to 30. This well-documented phenomenon can progress to agranulocytosis (absolute neutrophil count < 0.1 × 10⁹/mL), increasing the risk of sepsis and mortality. Neutropenia has been linked to immune-allergic or toxic mechanisms and described as a “maturation arrest”, where neutrophils are retained in the bone marrow and fail to enter peripheral blood. Antibodies against the benzylpenicilloyl determinant are also more common in patients with DHR [11,12,13,14].

Symptoms of DHR include fever, rash, low neutrophil count, and infusion-related discomfort. Although DHR can occur with any beta-lactam, it is more frequent with penicillin G, oxacillin, and certain cephalosporins. Reported incidence ranges from 10% to 30%, emphasizing the need for close monitoring during prolonged treatment. DHR is exceptionally rare in patients on oral therapy [11,12].

In the context of IE treatment, there is limited knowledge regarding the optimal plasma concentrations in relation to MIC and the time that the plasma concentration of the drug remains over the MIC of the targeted bacteria (T > MIC). Plasma levels have been described to have large intra-individual variance in previous studies including continuous infusion of antibiotics in IE with focus on ampicillin [15,16,17,18].

The primary objectives of this study were to measure plasma concentration of the two most used beta-lactam antibiotics in the treatment of IE in Sweden—benzylpenicillin and cloxacillin—and to describe inter- and intra-individual variations in these concentrations. Furthermore, the study aimed to explore factors explaining the variations in plasma concentrations and their potential association with DHR.

## 2. Results

### 2.1. Baseline Characteristics and Demographics

**Figure 1:**
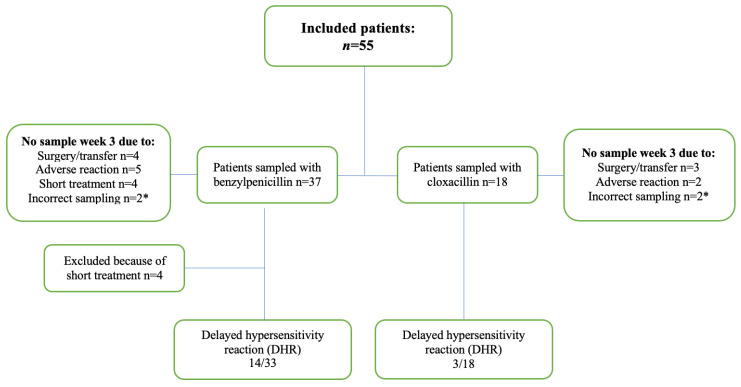
Flowchart of the study. * Missing single sample.

In total, 55 patients were included in the study and 184 plasma samples were collected. Complete sampling series, comprising two samples (centre and trough) during the first and third weeks, were obtained for 38 patients. Samples from the third week were missing in 18 patients (Figure 1).

The reasons for missing samples included short IE treatment duration (four patients), change of treatment due to hypersensitivity reactions (seven patients), and surgery or referral to another hospital (seven patients). Finally, four individual samples were excluded from the analysis due to incorrect sampling time. None of the samples fell outside the analytical range of the method (0.1–100 mg/L). The patients’ demographic data are summarized in Table 1.

A majority of the patients (37/55, 67%) received treatment with benzylpenicillin. All subjects, regardless of the specific type of penicillin (benzylpenicillin or cloxacillin), received the same dose as defined by the inclusion criteria (3 g every 6 h). Thirty-eight (69%) patients had definite IE, and a majority had left sided native valve involvement. The remaining 17 cases were classified as possible endocarditis based on the same criteria. The causative bacteria were mainly viridans group streptococci in patients treated with benzylpenicillin, and *Staphylococcus aureus* in patients treated with cloxacillin (Supplementary Table S1). Cardiac surgery during treatment was numerically more frequent in individuals with staphylococcal IE (7/18, 39%) compared to in patients with streptococcal IE (8/37, 22%), the difference, however, not reaching statistical significance. In-hospital and 90-day mortality was 3% (2/55) while mortality at one year was 9% (5/55).

### 2.2. Plasma Concentrations

**Figure 2:**
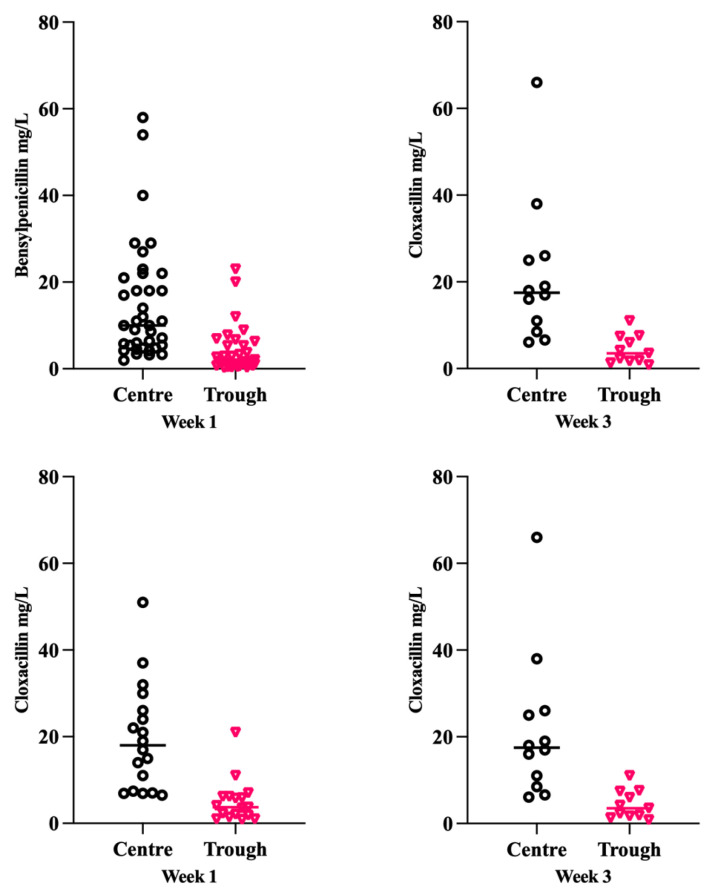
Centre and trough total plasma concentrations of benzylpenicillin and cloxacillin in treatment weeks 1 and 3. Benzylpenicillin, median (IQR); centre value, median 10 (5.1–21.5) and 20 (6.5–29.0), trough value 1.6 (0.79–5.3) and 3.6 (1.1–6.8). Cloxacillin, median (IQR); centre value 18 (7.3–27.0) and 17.5 (9.1–25.8). Trough value 3.7 (1.7–6.2) and 3.85 (1.7–7.4).

Overall, the administration of both benzylpenicillin and cloxacillin yielded similar plasma concentrations. However, there was considerable interindividual variation; benzylpenicillin centre values varied between 1.9 and 67 mg/L, and trough values between 0.19 and 33 mg/L. For cloxacillin, centre values varied between 6.1 and 66 mg/L, and trough values between 0.83 and 21 mg/L, as shown in Figure 2.

**Figure 3:**
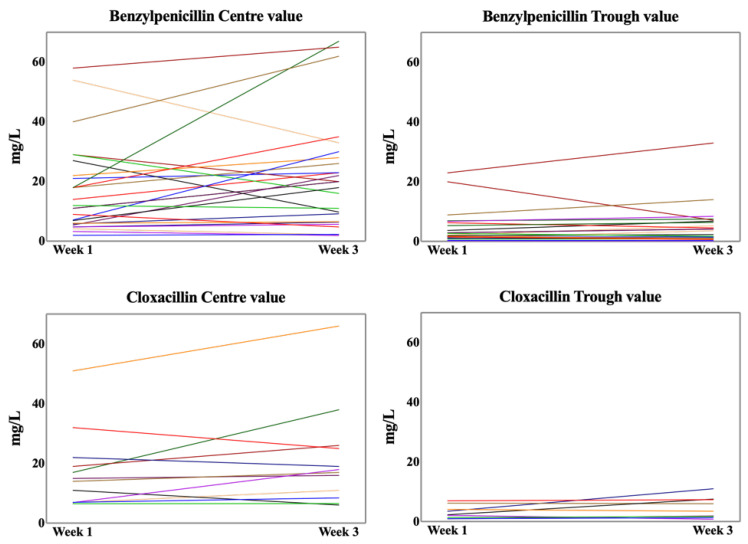
Paired plasma concentrations (treatment weeks 1 and 3) of benzylpenicillin and cloxacillin. Wilcoxon signed-rank test with no significant intra-patient difference between weeks. Effective pairing with Spearman (one-tailed) 0.7–0.9, *p* < 0.01.

The intra-individual variation between week one and three was small for most patients, but a few individuals showed larger changes in concentration over time (Figure 3). Spearman correlation coefficients calculated for the individually paired samples ranged from 0.7 to 0.9 (*p* < 0.01).

In the univariate analysis of benzylpenicillin concentrations, significant associations were found between trough plasma concentrations and age, as well as with creatinine levels (Table 2).

In a multivariate linear regression model including variables with univariate *p*-values < 0.25 (age, creatinine, BMI, and albumin), the adjusted coefficient of determination (*R*^2^) for trough benzylpenicillin concentration was 0.68 (*p* < 0.001), indicating the degree of explanatory power for this model. In comparison, the coefficient of determination for absolute eGFR was 0.54 (*p* < 0.001). Within the more limited subset of patients treated with cloxacillin, only age had a significant univariate correlation with the trough concentration (*R*^2^ 0.26, *p* = 0.02). Integration into a multivariate model that also included plasma creatinine levels did not improve the degree of explanation *R*^2^ 0.21, (*p* = 0.072). Notably, univariate analysis using absolute eGFR calculated by the Lund–Malmö formula marginally outperformed age (*R*^2^ 0.30, *p* = 0.01), as shown in Table 2. For both benzylpenicillin and cloxacillin, correlations were similar if centre plasma concentrations were used in the models (Supplementary Table S2).

### 2.3. Delayed Hypersensitivity Reaction

In the analyses of DHR, four patients with short duration of treatment (14 days) were excluded. In total, 17 patients met the criteria for DHR; 14 out of 33 (42%) in the benzylpenicillin group compared to 3 out of 18 (17%) in the cloxacillin group. Symptoms of DHR included fever, low neutrophil count (<1.5 × 10^9^/L), rash, or infusion-related discomfort, as listed in Table 3.

Among the 17 patients treated with benzylpenicillin who experienced DHR, centre and trough plasma concentrations were significantly lower compared to patients receiving the same antibiotic who did not experience DHR, as shown in Table 4. This difference was more pronounced in the subgroup with low neutrophil counts. Consistent with this finding, patients with DHR had higher absolute eGFR, with a median of 93 mL/min (IQR: 79–109), compared to 77 mL/min (IQR: 63–100) in the non-DHR group (*p* = 0.04).

Patients who developed DHR tended to be younger, with a median age of 63 years (IQR: 51–68), compared to 75 years (IQR: 52–81) in the non-DHR group (*p* = 0.06). No significant differences were observed between the DHR and non-DHR groups in terms of BMI or sex. Among the patients with DHR, altogether nine individuals developed a low neutrophil count (≤1.5 × 10^9^/L) during treatment, with six patients progressing to neutropenia (≤1.0 × 10^9^/L). In patients with DHR, the shift of antibiotic due to DHR was performed on median day 21 of treatment (range day 13–28). Analysis of the DHR cloxacillin group was not performed due to the small sample size.

## 3. Discussion

In this study, we investigated plasma concentrations of benzylpenicillin and cloxacillin in patients undergoing treatment for infective endocarditis (IE) using standard doses (12 g per day). Our findings revealed a wide range of plasma concentrations, with interpatient variations of 30- to 100-fold in both trough and centre levels. Importantly, no patients exceeded toxic levels, a reassuring result supported by previous studies and expert opinion [19,20,21]. As expected, variations in antibiotic concentration were strongly correlated with kidney function and age, consistent with pharmacokinetic principles. Modest associations were also observed between plasma concentrations and BMI, as well as albumin levels, indicating that body composition and protein-binding capacity can also influence drug distribution, while no correlation was found with sex. Higher BMI and albumin levels were associated with lower plasma concentrations of benzylpenicillin in our multivariate model. This relationship was not observed for cloxacillin, possibly due to the small sample size for that subgroup. In the benzylpenicillin group, there was a tendency for higher median values in week 3 of treatment. This might reflect several factors, including the potential loss of patients in sampling during week 3 due to short treatment or surgical intervention.

The multivariate models developed in this study explained 68% of the plasma concentration variability for benzylpenicillin and 21% for cloxacillin plasma concentrations in IE patients. This proportions aligns closely with the explanatory power of standardized formula to calculate total glomerular filtration rate. The Lund–Malmö eGFR equation used in our study explained 54% of the plasma concentration variations for benzylpenicillin and 30% for cloxacillin. This consistency suggests that GFR, calculated without adjustment for body surface area, plays a substantial role in determining beta-lactam levels in the bloodstream. However, the remaining unexplained variability in penicillin concentrations, often termed “true variability”, which cannot be explained by known factors, raises the potential role of therapeutic drug monitoring (TDM). This variability underscores the potential utility of TDM in specific patient populations with unpredictable pharmacokinetics, such as those with extreme obesity or renal dysfunction. Although the study lacks the power to draw definitive conclusions, particularly for patients treated with cloxacillin, the findings cautiously suggest that routine TDM may have a less significant role in clinically stable IE patients.

To our knowledge, this is the first prospective study to evaluate penicillin concentrations in patients with DHR. It is well established that long-term, high-dose penicillin can trigger neutropenia, with neutrophil counts typically normalizing within a week after discontinuation of the causative penicillin. The pathogenesis of penicillin-related DHR remains unclear and may involve multiple mechanisms. In our study, 33% of patients treated with penicillin for 14 days or more exhibited symptoms of DHR, a frequency consistent with previous findings [12]. Antibiotics are often switched early at the onset of any symptom consistent with DHR, thus preventing the development of a more severe and protracted reaction. We defined “low neutrophil count” as a number below the local reference threshold (<1.5 × 10^9^/L), but in fact all patients in this group had neutrophil counts below 1.25 × 10^9^/L. Notably, we found no association between elevated penicillin concentrations and DHR. Interestingly, patients with low neutrophil counts exhibited lower antibiotic levels, raising the possibility that factors such as younger age or better renal function may influence this finding. However, the observed association between neutropenia and lower serum levels warrants further investigation to determine whether beta-lactam pharmacokinetics, such as tissue penetration or bone marrow exposure, contribute to this effect. Other pharmacodynamic parameters, like the area under the inhibitory curve (AUIC), could provide a broader understanding of tissue distribution and offer insights into these mechanisms.

The association between lower antibiotic concentrations and DHR observed in our study was unexpected. While higher doses over time are typically assumed to pose a greater risk for DHR, our findings suggest that the pathogenesis involve a more nuanced interplay between total drug exposure, plasma concentrations, immune response, and patient characteristics, resulting in an idiosyncratic reaction. This aligns with previous research indicating that immune-mediated reactions to beta-lactams may not solely depend on dose but rather on individual patient factors, including genetic predisposition, immune status, and drug metabolism pathways.

It has been suggested that high serum concentrations may be associated with the development of neutropenia during beta-lactam therapy [22]. However, our findings do not support this assumption. In this study, patients who developed neutropenia did not exhibit high serum penicillin levels. This suggests that it is sustained high exposure over time, rather than absolute serum concentrations, that are critical in the development of this adverse effect. This suggests that TDM-guided adjustments are unlikely to predict or prevent neutropenia. Future studies could aim to investigate specific immune markers or genetic factors that could help predict which patients are at higher risk for DHR when exposed to beta-lactam antibiotics.

Several limitations of this study should be considered when interpreting the results. First, the sample size was relatively small, which may affect both internal and external validity of the findings. The limited sample size could have reduced the statistical power of the study, particularly in the analyses involving cloxacillin, where associations between plasma concentrations and patient variables were harder to establish. Additionally, the patient group consisted of clinically stable IE patients, which may not fully represent the broader IE population. More severely ill patients, especially those with infections caused by *Staphylococcus aureus*, were underrepresented, contributing to the low observed mortality rates. This selection may limit the generalizability of the results to patients with higher disease severity or infections caused by different pathogens. Another limitation was that we measured total penicillin concentrations rather than the free, microbiologically active fraction, which was unavailable for clinical use at the time of the study. This is of particular interest for cloxacillin, which is highly bound to plasma proteins in the circulation. Finally, the decision to discontinue penicillin due to DHR was made by the attending physician and retrospectively confirmed.

## 4. Materials and Methods

### 4.1. Design and Setting

This prospective observational study was conducted between May 2017 and April 2020, at the departments of Infectious Diseases in three hospitals in southwest Sweden: Sahlgrenska University Hospital, Southern Älvsborgs Hospital, and Northern Älvsborgs Hospital.

Patients eligible for inclusion were those aged 18 years or older who were receiving treatment for suspected or confirmed IE, as defined by the modified Duke criteria at any of the three participating sites. The Duke criteria for infective endocarditis (IE) use clinical, microbiological, and echocardiographic findings, including transesophageal echocardiography (TEE), to classify cases as definite, possible, or rejected. Major criteria include positive blood cultures and endocardial involvement (e.g., vegetations on TEE), while minor criteria cover risk factors, fever, vascular or immunological phenomena [23].

The patients were receiving treatment with benzylpenicillin or cloxacillin intravenously at standard doses in accordance with Swedish IE guidelines [7]. A standard dose was defined as 3 g every 6 h. Patients with reduced antibiotic dosing due to body surface area-adjusted estimated glomerular filtration (eGFR) below 30 mL/min/1.73 m^2^ were excluded. Cloxacillin was administered as a bolus infusion over 30 min, and benzylpenicillin as an intravenous injection. Two blood samples were drawn at 3 h after dose administration (centre concentration) and again at 6 h just before next dose (trough concentration) during the first and the third week of treatment. The samples that deviated more than +/− 15 min from scheduled sampling time were excluded. The samples were stored at 8 °C and within 24 h centrifuged and thereafter frozen at −80 °C until analysis. Samples were analysed at the Department of Clinical Biochemistry, Sahlgrenska University Hospital, Gothenburg, using liquid chromatography coupled to tandem mass spectrometry [24], providing total antibiotic concentrations in plasma. The assay had been validated according to the approved guidelines issued by the Clinical and Laboratory Standards Institute (CLSI, ISO15189 [25]). In the analyses of DHR, four patients with short duration of treatment (14 days) were excluded.

### 4.2. Ethics

Ethical approval was obtained from the Regional Ethical Committee (ID702116) and the study protocol was in accordance with the Declaration of Helsinki. Written informed consent was obtained from each patient participating in the study.

### 4.3. Data and Definitions

Demographic and clinical data, including laboratory results, were prospectively collected in a standardized case report form. All treatment decisions, including modifications of antibiotic regimens, were made by the attending physicians and were not influenced by the study investigators. Absolute eGFR was calculated with the Lund–Malmö-formula, LMR18/LM-Rev-equation [26]. DHR was diagnosed by the attending physicians in presence of one or several of following symptoms; recurrent fever, low neutrophil count (<1.5 × 10^9^/L), rash, and infusion related discomfort. It was retrospectively confirmed by those responsible for the study based on patients’ medical records. Symptoms were considered as drug induced if they met the following criteria: (1) occurred following antibiotic administration, (2) ceased upon drug withdrawal, and (3) no other more probable explanation for the described symptoms.

### 4.4. Microbiological Analysis

Blood samples were processed using aerobic and anaerobic blood culture bottles (40 mL/set) with growth media (BacT/Alert, bioMérieux, Marcy l’Etoile, France). Cultures were incubated in the BacT/Alert 3D system for up to 10 days following standard procedures. Positive cultures were subcultured onto agar plates and incubated overnight in a CO_2_-rich atmosphere at 35 ± 2 °C. Bacterial identification was performed using MALDI-TOF MS (VITEK MS PRIME, bioMérieux, Marcy l’Etoile, France). Antimicrobial susceptibility testing followed the European Committee on Antimicrobial Susceptibility Testing (EUCAST) guidelines [27], with results reported as susceptible, intermediate, or resistant (SIR). Negative cultures after 10 days were recorded as such. All steps adhered to strict aseptic techniques to prevent contamination.

### 4.5. Statistics

The Mann–Whitney U-test, Wilcoxon signed rank test, Chi^2^ test, Fisher’s exact test, and the Spearman correlation coefficient test were used as appropriate. Analyses were performed using SPSS Version 29 (IBM SPSS Statistics for Windows, Armonk, NY, USA) and PRISM Version 9 (GraphPad Software, San Diego, CA, USA). Statistical significance was considered if the two-sided *p* value was less than 0,05. Multivariate logistic regression, including variables with a univariate *p* value of less than 0.25, was preformed to analyse the association between patient factors concentration. This analysis was conducted with RStudio 2023.03.0.

## 5. Conclusions

In conclusion, our study highlights significant interindividual variability in plasma penicillin concentrations among IE patients. While TDM may not be beneficial for most patients on standard dosing, it could be useful in cases with unpredictable plasma concentrations. Our findings also challenge the assumption that TDM is a tool to prevent DHR and neutropenia, supporting the role of immunological factors. Further research is needed to better understand the factors driving variability and to identify patient profiles that might benefit from personalized dosing strategies or TDM. Despite its limitations, this study advances our understanding of penicillin pharmacokinetics in IE treatment and emphasizes the complexity of drug concentration-response relationships in clinical practice.

## Figures and Tables

**Table 1:** Baseline characteristics and outcomes.

	All *n* = 55 (%)	Benzylpenicillin *n* = 37 (%)	Cloxacillin *n* = 18 (%)
Sex, male	37 (67)	26 (70)	11 (61)
Age, median (IQR)	64 (53–79)	65 (53–82)	64 (47–74)
BMI ^1^, median, (min-max)	26 (18–41)	25 (18–38)	28 (18–41)
Comorbidities			
Coronary heart disease	10 (18)	7 (19)	3 (17)
Congestive heart failure	9 (16)	5 (14)	4 (22)
Diabetes mellitus	6 (11)	2 (5)	4 (22)
Cerebrovascular lesion	4 (7)	3 (8)	1 (11)
Pulmonary disease	8 (15)	6 (22)	2 (22)
Rheumatic disease	2 (4)	1 (3)	1 (11)
CCI—score ^2^, median (IQR)	1 (0–2)	1 (0–2.5)	1 (1–2)
Previous endocarditis	8 (15)	7 (19)	1 (11)
Duke modified criteria			
Definitive	38 (69)	25 (68)	13 (72)
Possible	17 (31)	12 (32)	5 (28)
Native valve endocarditis *	34 (62)	20 (54)	14 (78)
Mitral valve	14 (25)	12 (32)	2 (11)
Aortic valve	15 (27)	10 (27)	5 (28)
Tricuspid valve	8 (15)	1 (3)	7 (39)
Prosthetic valve endocarditis	16 (29)	15 (41)	1 (6)
CIED ^3^ endocarditis	7 ** (13)	2 (5)	5 ** (28)
Embolism, n (%)	25 *** (45)	15 (41)	10 *** (56)
Central nervous system	6 (11)	6 (16)	–
Pulmonary embolism	5 (9)	–	5 (28)
Peripheral artery	3 (5)	3 (8)	–
Spondylodiscitis	8 (15)	5 (14)	3 (17)
Other	4 (7)	1 (3)	3 (17)
Lab results (at time of study inclusion)			
Creatinine (μmol/L), median (IQR)	81 (66–90)	79 (66–88)	81 (65–105)
NT-pro BNP (ng/L), median (IQR)	1580 (246–3870)	1370 (246–4050)	1930 (207–3577)
Albumin (g/L), median (IQR)	31 (26–33)	32 (27–34)	28 (21–33)
Neutrophil count (10^9^/mL), median (IQR)	5.8 (4.1–7.8)	6.0 (4.1–7.6)	5.5 (3.9–10.6)
CRP (μmol/L), median (IQR)	49 (20–89)	48 (21–71)	73 (18–176)
CRP (μmol/L), admission, median (IQR)	76 (41–140)	69 (39–91)	145 (68–254)
Heart valve surgery during admission	14 (25)	8 (22)	7 (39)
Days to surgery, median (IQR)	12.5 (10–22)	13 (10–19)	12 (11–34)
Antibiotic treatment duration, median (IQR)	28 (26–41)	29 (2.5–41)	27.5 (26–32)
Short treatment (14 days)	4 (7)	4 (11)	–
Concomitant aminoglycoside	28 (76)	28 (76)	–
Mortality			
In-hospital	2 (4)	1 (3)	1 (6)
90 days	2 (4)	1 (3)	1 (6)
1 year	5 (9)	3 (8)	2 (11)

^1^ Body mass index, weight/height [height^2^ (kg/m^2^)]; ^2^ Carson comorbidity index; ^3^ cardiac implantable electronic device, * 3 patients with combined valve engagement. ** 5 patients with isolated CIED infection, *** 1 patient with two manifestations (pulmonary embolism and spondylodiscitis).

**Table 2:** Univariate and multivariate regression analyses of variables associated with trough plasma concentrations of benzylpenicillin and cloxacillin.

	Univariate			Multivariate			
**Benzylpenicillin (trough concentration) *n* = 37**							
	β	*r* ^2^	*p*	β	*r* ^2^	*p* (variable)	*p* (model)
Age	0.15	0.31	<0.001	0.09	0.68	0.004	<0.001
Creatinine (plasma)	0.17	0.44	<0.001	0.15		<0.001	
BMI *	−0.34	0.05	0.10	−0.21		0.03	
Albumin (plasma)	−0.28	0.06	0.08	−0.21		0.04	
Gender	0.86	−0.02	0.65	-	-	-	
Absolute eGFR **	−0.15	0.54	<0.001				
**Cloxacillin (trough concentration) *n* = 18**							
	β	*r* ^2^	*p*	β	*r* ^2^	*p* (variable)	*p* (model)
Age	0.19	0.26	0.02	0.17	0.21	0.06	<0.001
Creatinine (plasma)	0.06	0.05	0.19	0.02		0.64	
BMI *	−0.02	−0.07	0.94	-	-	-	
Albumin (plasma)	−0.01	−0.06	0.99	-	-	-	
Gender	2.2	−0.01	0.38	-	-	-	
Absolute eGFR **	−0.13	0.30	0.01				

β: regression coefficient; *r*^2^: adjusted coefficient of correlation. * Body Mass Index; ** absolute estimated glomerular filtration rate (eGFR) calculated by the Lund–Malmö formula.

**Table 3:** Symptoms of delayed hypersensitivity reaction.

	All Patients (*n* = 51)	Benzylpenicillin (*n* = 33)	Cloxacillin (*n* = 18)
Delayed hypersensitivity reaction	17	14	3
Low neutrophil count *	9 **	7	2
Fever	7	7	
Rash	3	2	1
Infusion-related discomfort	2	2	

* Below local reference point 1.5 × 10^9^/L. ** 6 patients with manifest neutropenia ≤ 1.0 × 10^9^/L at diagnosis of delayed hypersensitivity reaction.

**Table 4:** Plasma benzylpenicillin concentration in patients with and without delayed hypersensitivity.

	No Delayed Hypersensitivity Reaction (*n* = 19)	Delayed Hypersensitivity Reaction (*n* = 14)	*p*
Centre value (mg/L)	18.6 (13.7–25.0)	6.6 (4.0–13.2)	0.04
Trough value (mg/L)	3.6 (1.5–5.9)	0.94 (0.4–2.3)	0.0053
	**Normal neutrophil count** **(*n* = 26)**	Low neutrophil count ** **(*n* = 7)**	
Centre value (mg/L)	18.5 (10.0–23.5)	3.4 (1.5–6.2)	0.0008
Trough value (mg/L)	5.4 (3.2–7.5)	0.8 (0.4–1.7)	0.0141

Plasma benzylpenicillin concentration, individual plasma concentration calculated as the mean of week 1 and 3 in each patient, or, if single sample, shown as median and interquartile range. ** Below local reference point 1.5 × 10^9^/mL.

## Data Availability

The data underlying this article will be shared on reasonable request to the corresponding author.

## Supplementary Materials

The following supporting information can be downloaded at: https://www.mdpi.com/article/10.3390/antibiotics14010056/s1, Table S1: Microbiological etiology and antibiotic sensitivity; Table S2: Univariate and multivariate regression analyses of variables associated with centre plasma concentrations of benzylpenicillin and cloxacillin.
