# Supplementary material for: Plasma Concentrations of Benzylpenicillin and Cloxacillin in Infective Endocarditis—With Special Reference to Delayed Hypersensitivity Reactions
Source: Antibiotics (Basel). 2025 Jan 9;14(1):56. doi: 10.3390/antibiotics14010056 (PMC11763166; doi:10.3390/antibiotics14010056)
Supplement: Supplementary file 1 [file antibiotics-14-00056-s001.zip › antibiotics-3380955-supplementary.pdf]

Supplementary Materials

Table S1. Microbiological etiology and antibiotic sensitivity.

Microorganism	Number of patients	MIC[1] range (mg/L)
Staphylococci	19	
Staphylococcus aureus	18	<2*
Staphylococcus epidermidis	1	<2*
Streptococci	34	
Viridans group**	28	0.016-0.25
Group B streptococci	2	0.064-0.125
Nutritionally variant***	4	0.032-0.19
Culture negative	2	

[1]Minimal Inhibitory Concentration; **Oxacillin; **Streptococcus mitis-group (18), Streptococcus sanguinis (4), Streptococcus bovis (3), Streptococcus anginosus (1), Streptococcus mutans (1), Streptococcus salivarius(1); ***Granulicatella adiacens (2) Abiotrophia defectiva (2)*

Table S2. Univariate and multivariate regression analyses of variables associated with ***centre*** plasma concentrations of benzylpenicillin and cloxacillin.

	Univariate			Multivariate			
	β	r^2	p	β	r^2	p (variable)	p (model)
Benzylpenicillin (Centre value) n=37							
Age	0.37	0.27	<0.001	0.21		0.012	
Creatinine (plasma)	0.41	0.37	<0.001	0.38	0.63	<0.001	<0.001
BMI*	-0.95	0.07	0.10	-0.80		0.03	
Albumin (plasma)	-0.61	0.03	0.15	-0.44		0.13	
Gender	5.64	0.01	0.07	-	-	-	
Absolute eGFR**	-0.43	0.59	<0.001				
Cloxacillin (Centre value) n=18							
Age	0.48	0.35	0.006	0.44	0.40	0.01	<0.001
Creatinine (plasma)	0.17	0.11	0.19	0.12		0.15	
BMI**	0.08	-0.06	0.88	-	-	-	
Albumin (plasma)	0.15	-0.06	0.75	-	-	-	
Gender	5.37	-0.01	0.38	-	-	-	
Absolute eGFR**	-0.39	0.47	0.001				

β: regression coefficient. r^2: adjusted r-squared (coefficient of correlation).
*Body Mass Index, ** estimated glomerular filtration rate, calculated by the Lund/Malmö formula.
